# Clinical significance of serum antinuclear antibodies in patients with autoimmune hepatitis and comorbidity

**DOI:** 10.1007/s10238-023-01094-1

**Published:** 2023-05-23

**Authors:** Hui Liu, Yuqi Wang, Peng Wang, Xin Wang, Yunyun Yi, Xin Li

**Affiliations:** 1 Department of Center of Integrated Traditional Chinese and Western Medicine, Beijing Ditan Hospital, Capital Medical University, Beijing, China; 2 Department of Center of Integrated Traditional Chinese and Western Medicine, Peking University Ditan Teaching Hospital, Beijing, China

**Keywords:** Autoimmune hepatitis, Immune disease, ANA, Prognosis

## Abstract

Autoimmune hepatitis (AIH) is often complicated with immune diseases, which greatly affected the course and clinical outcome of AIH. We aimed to systematically assess clinical characteristics, prognosis in autoimmune hepatitis accompanied by immune diseases. Clinical records of 358 patients with AIH from Beijing Ditan Hospital in China were retrospectively reviewed. The clinical features of AIH with immune diseases were compared retrospectively, including clinical characteristics, prognosis and outcome. Prevalence of immune diseases in patients with AIH was 26.5%. Connective tissue disease (CTD) was the commonest immune diseases associated with AIH (33/358, 9.2%), and the incidence of primary biliary cholangitis (PBC) and thyroid dysfunction (TD) was low (4.7% and 8.5%, respectively). At diagnosis, AIH-PBC patients had higher IgM and ALP, lower weight, Hgb, ALT and AFP (*P* < 0.05). Meanwhile, AIH-CTD patients had lower mean platelet volume, serum K and triglyceride (*P* < 0.05). AIH-TD patients had a lower proportion of ANA positive (*P* < 0.05). The overall survival time of AIH-TD was significantly shorter than AIH patients (*P* = 0.0011), but there were no differences in AIH-PBC and AIH-CTD. Furthermore, ANA negative (*HR*: 0.21, 95%*CI* 0.13–0.35, *P* < 0.001) can be a factor to predict the poor prognosis of AIH, and also in AIH-TD patients. About 26.5% of AIH patients had at least one immune disease, and TD coexisted with AIH impaired patients’ survival. ANA negative can be used as an independent indicator to predict the poor prognosis of AIH and AIH-TD.

## Introduction

Autoimmune hepatitis (AIH) is an immune-mediated inflammatory diseases of hepatic parenchymal cells [1]. Its pathogenesis is complex, which is different from viral infection, alcohol injury and fat deposition [2]. AIH is mainly manifested as interface hepatitis infiltrated by lymphocytes and plasma cells, resulting in damage of liver parenchyma cells, subsequent fibrosis, cirrhosis and eventually liver failure [3, 4].

Most patients with AIH are of young women initially, but since the late 1990s, more and more studies reported that AIH could occur in different age, even in over 60 years old [5, 6]. Some studies have also found that AIH can be found in different geographical regions, including Europe, the North America, China, India and New Zealand [7]. In recent years, the rising incidence of AIH has aroused widespread concern. It was reported that the contemporary incidence rate in European countries increases from 0.4/100,000 to 2.39/100,000 [8]. A prospective study from New Zealand showed that the incidence of AIH increased by 1.93/100,000 from 2008 to 2016 [9]. Similarly, a recent study from the US reported an AIH prevalence as high as 31.2/100,000 based on a commercial database from 2014 to 2019 [10]. All above studies indicated that AIH was distributed in people of any age and race. Although AIH is relatively rare, the clinical burden is disproportionately high compared with the morbidity and prevalence of the population [11], which brings a serious burden to the patients.

The most common manifestations of AIH are elevated serum aminotransferase level, hyper IgG and positive serum autoantibodies [12]. In view of the fact that AIH is mainly caused by immune-mediated liver diseases, it can involve multiple organs throughout the body. It is shown that about 30–35% of AIH patients have extrahepatic immune diseases, such as primary sclerosing cholangitis (PSC), thyroiditis, Sjogren's syndrome and other immune diseases [13]. A study from Italy showed that about 42% of AIH patients were complicated with extrahepatic immune diseases [14]. Notably, it is presented that about 50% of children and young patients had overlapping phenotype in the west [15]. The coexistence of immune diseases makes the diagnosis and treatment of AIH more difficult, and affects the prognosis and the life quality of patients. The coexistence of such diseases often increases the difficulty of AIH diagnosis and treatment.

In view of the above situation and the lack of prognostic factors related to AIH and AIH complication, we collected the clinical characteristics of AIH and comorbidity in China from a clinical and research center for liver disease. In this research, a formed database was established, which records AIH patients admitted in Beijing Ditan Hospital since 2008, and these are the main data resources of the research. The clinical features of AIH complicated immune diseases including baseline characteristics, overall survival and vital status were analyzed, respectively.

## Materials and methods

### Study population

A total of 358 consecutive patients, who had been diagnosed with AIH from October 2008 to May 2020 at Beijing Ditan Hospital, were included in this study. All patients were negative for hepatitis B or C infections. Patients who were heavy alcohol drinkers (> 25 g/day of alcohol), with Wilson’s disease or nonalcoholic fatty liver disease, with extrahepatic biliary disease, chronic liver disease other than AIH, taking drugs with known hepatotoxicity and a history of malignancy within 5 years were excluded. Patients with incomplete data were also excluded. This study was approved by the Ethics Committee of Beijing Ditan Hospital.

### Diagnosis of AIH

For the diagnosis of AIH, both the revised IAIHG criteria and the simplified IAIHG criteria were used [16, 17]. If a patient met any of the criteria, then the patient was determined as a probable or definite AIH patient; at least 10 points for the revised criteria and 6 points for the simplified criteria.

### Definition of immune diseases

All patients were evaluated for the presence of immune diseases and assessed systemic involvement. PBC was diagnosed based on the American Association for the Study of Liver Diseases (AASLD) 2009 Practice Guidelines [18]. The diagnostic criteria of Sj¨ogren’s syndrome (SS) was based on the LeRoy classification [19]; The diagnosis of rheumatoid arthritis (RA) was made on the basis of revised classification criteria of the American College of Rheumatology [20]; Systemic Lupus Erythematosus (SLE) was diagnosed according to the revised ACR SLE classification criteria of the Systemic Lupus Collaborating Clinics (SLICC; 2009) [21]. The SS, SLE and RA were classified as connective tissue disease (CTD). Thyroid dysfunction (TD) was based on the combination of clinical features, the presence of serum antibodies against thyroid antigens (mainly TSH, TPOAb and TGAb) and thyroid sonography [22].

### Data collection and follow-up

Clinical data and quality indicators related to laboratory testing were obtained through inpatient medical record system. Survival time was determined by follow-up. All patients were followed up by telephone or in-hospital follow-up review until death appears or until December 31, 2021, whichever comes first.

### Statistical analysis

Data was analyzed by using the SPSS statistical software (Version 19.0. IBM Corp, Armonk, NY, USA) and R Studio software (Version 3.5.1).

Continuous variables were expressed as mean ± standard deviation (SD) or median (range) and categorical variables were presented as frequencies or ratios. Nonparametric variables were presented as median with interquartile range. Comparison of outcomes between groups was performed by using Student’s t-test or Mann–Whitney U test for continuous data; statistical significance for categorical data was determined by using the Chi-square test or Wilcoxon rank-sum test. Univariate and multivariate cox regression analysis was used to determine the independent influencing factors related to AIH prognosis, and the hazard ratio (HR) and 95% confidence interval (CI) of the prognostic factors were shown in the forest map. Kaplan–Meier method was used to analyze the survival of AIH and comorbidity. All statistical tests were two-sided, and *P* < 0.05 was considered statistically significant.

## Results

### Clinical characteristics

A total of 358 consecutive patients with AIH were enrolled from Beijing Ditan Hospital between October 2008 and May 2020. At diagnosis, the average age of AIH patients was about 55 years old. Among the 358 consecutive AIH patients, 81 patients complicated with one immune disease and 14 patients complicated with over two immune diseases. According to the types of immune diseases, it is mainly divided into four groups: AIH group (n = 277), AIH-PBC group (n = 17), AIH-CTD group (n = 33) and AIH-TD group (n = 31). Basic demographic information is presented in Table 1. Actually, the AIH-CHD group (9%) has the highest proportion in AIH complicated with immune diseases, followed by AIH-TD group (8.6%), and it is interesting that hypothyroidism (4.7%) accounts for the largest proportion in AIH-TD group. Therefore, AIH patients complicated with immune diseases are common, especially CTD and TD.

**Table 1:** AIH patients complicated with immune diseases

Comorbidities	Number of AIH patients, n (%)
Primary biliary cholangitis (PBC)	17 (4.7%)
Connective tissue disease (CTD)	33 (9.0%)
Sjogren’s syndrome	13 (3.6%)
Systemic lupus erythematosus	5 (1.3%)
Rheumatoid arthritis	8 (2.2%)
Overlapping ctd	7 (1.9%)
Thyroid dysfunction (TD)	31 (8.5%)
Graves’s thyroiditis	3 (0.8%)
Hashimoto’s thyroiditis	3 (0.8%)
Hypothyroidism	17 (4.7%)
Thyroid nodule	8 (2.2%)

The baseline characteristics of the patients with AIH, AIH-PBC, AIH-CTD and AIH-TD is presented in Table 2. Interestingly, it is shown that the weight, ALT and AFP levels in AIH group are higher than AIH-PBC group (*P* = 0.01, *P* = 0.028 and *P* = 0.034, respectively), while IgM and ALP levels are significantly lower (*P* = 0.044 and *P* = 0.003, respectively). In addition, it is also found that AIH-CTD patients showed a lower level of serum K, MPV and TG (*P* = 0.029, *P* = 0.038 and* P* = 0.035, respectively). The proportion of ANA-positive in AIH-TD patients is significantly less than that of AIH patients (*P* = 0.02). None of the patients have a positive test for LKM-1.

**Table 2:** Baseline characteristics of the patients with AIH, AIH-PBC, AIH-CTD and AIH-TD

Characteristics	AIH (n = 277)	AIH-PBC (n = 17)	AIH-CTD (n = 33)	AIH-TD (n = 31)	*P-*value
Female	243/277(87.7%)	14/17(82.4%)	28/33(84.8%)	30/31(96.8%)	NS*†‡
Age (years)	56(48–64)	58(41.5–66)	55(48–62.5)	57(43–63)	NS*†‡
Weight (kg)	60(55–68)	55(48.38–58.25)	60(52.63–65.5)	60.5(55–67)	0.010*/NS†‡
IgG (g/L)	21.4(18.3–25.78)	22.6(16.4–34.3)	21.6(19.5–26.2)	21.4(18.75–29.25)	NS*†‡
IgA (g/L)	3.435(2.38–4.77)	4.45(2.92–6.25)	3.97(2.95–5.14)	3.45(2.28–4.30)	NS*†‡
IgM (g/L)	1.53(0.98–2.28)	2.71(1.46–3.8)	1.26(0.9–2.13)	1.28(0.97–2.09)	0.044*/NS†‡
WBC (10^9^/L)	4.52(3.37–5.88)	5.04(3.45–6.13)	4.17(3.38–5.9)	5.02(3.52–6.54)	NS*†‡
NE% (%)	54.32 ± 12.26	53.96 ± 18.71	55.95 ± 16.6	56.63 ± 11.08	NS*†‡
MO% (%)	9.8(7.58–12.38)	9.35(6.05–10.13)	9.93(7.45–12.06)	9.9(7.67–11.37)	NS*†‡
Hb(g/L)	118.6(103.28–131)	105(95–119.15)	112.9(101.25–125.85)	117.25(103.8–136.5)	0.028*/NS†‡
PLT (10^9^/L)	146(93.4–200.53)	95.8(60.25–206.5)	121.3(83.4–198.85)	165.7(120.03–217.25)	NS*†‡
MPV (fl)	10.1(9–11.4)	9.7(9.05–11.25)	9.3(8.8–10.61)	10.5(8.95–12)	NS*‡/0.038†
K (mmol/L)	3.61(3.35–3.89)	3.515(3.05–3.80)	3.37(3.09–3.84)	3.575(3.19–3.81)	NS*‡/0.029†
Na (mmol/L)	139.4(137.3–141.38)	138.45(135.78–141.15)	139.5(136.6–141.25)	139.9(138.68–140.65)	NS*†‡
Phos (mmol/L)	1.11(0.97–1.21)	1.205(0.95–1.29)	1.06(0.94–1.16)	1.15(1.04–1.27)	NS*†‡
UREA (mmol/L)	3.96(3.24–5.28)	4.28(3.46–5.17)	4.24(3.295–5.9)	4.065(3.19–4.60)	NS*†‡
CREA (umol/L)	57(50.05–65.65)	61.55(55.55–69.25)	58.2(52–69.5)	53.5(47.75–78.08)	NS*†‡
URCA (umol/L)	235(189–291)	195(150–284.5)	220(178.25–254.2)	224.5(159–293.75)	NS*†‡
GLU (mmol/L)	5.32(4.675–6.17)	5.22(4.425–6.84)	5.4(4.63–6.95)	5.09(4.75–6.23)	NS*†‡
ALT (U/L)	124.6(41.75–370.35)	61.4(25.7–109.7)	80.6(31.85–353.45)	162.8(58–354.6)	0.034*/NS†‡
AST (U/L)	134.2(56.75–327.5)	110(60.7–172.45)	98(41.35–373.2)	193.4(100.9–337.1)	NS*†‡
TBIL (umol/L)	43.25(16.73–109.68)	45.6(22.75–73.3)	31.9(15.45–92.6)	42.8(27.3–134.3)	NS*†‡
DBIL (umol/L)	27.8(8.2–85.2)	34.1(13.1–49.8)	20.9(6.75–62.4)	26.5(12.55–110.6)	NS*†‡
ALB (g/L)	33.61 ± 5.45	32.96 ± 7.3	32.28 ± 4.94	33.03 ± 5.0	NS*†‡
GLO (g/L)	39.1(35.95–44)	41.9(34.8–53.65)	39.9(35.9–48.5)	37.3(34.7–44.8)	NS*†‡
GGT (U/L)	147.7(61.2–274.7)	154.95(84.78–530.3)	139.75(63–233.28)	103.2(74.9–246.15)	NS*†‡
ALP (U/L)	130.2(90.9–195.4)	246.95(129.55–789.5)	113.5(87.83–257.08)	119.9(86.15–177.75)	0.003*/NS†‡
TBA (umol/L)	54.9(19–146.45)	88.3(24.28–130.98)	32.95(9.33–127.3)	80.6(18.5–183.55)	NS*†‡
TC (mmol/L)	3.76(3.07–4.35)	3.635(2.22–4.94)	3.32(2.91–4.045)	3.5(2.63–4.61)	NS*†‡
TG (mmol/L)	1.29(0.84–1.81)	1.13(0.69–1.96)	0.96(0.75–1.32)	1.17(0.85–2.46)	NS*‡/0.035†
HDLC (mmol/L)	0.71(0.32–1.03)	0.84(0.55–1.10)	0.91(0.48–1.11)	0.56(0.31–0.96)	NS*†‡
LDLC (mmol/L)	2(1.52–2.53)	2.225(1.41–3.74)	1.8(1.31–2.28)	2.09(1.32–2.43)	NS*†‡
PT (s)	13.1(11.9–15)	12.9(11.55–14.6)	12.95(11.2–15.55)	12.4(11.35–14.7)	NS*†‡
APTT (s)	35.4(32.6–39.75)	38.7(34.3–41.55)	35.7(31.55–42.15)	34.25(31.7–36.83)	NS*†‡
INR	1.13(1–1.29)	1.09(1–1.27)	1.14(0.98–1.34)	1.12(0.99–1.26)	NS*†‡
TT (s)	18.55(16.55–19.8)	18.7(16.4–19)	18.5(16.45–20.4)	18.4(16.8–20.28)	NS*†‡
AFP (ng/mL)	6.8(3.8–23.85)	4.4(2.6–5.2)	5.5(3.65–38.65)	7.9(3.93–22.36)	0.003*/NS†‡
ANA +	232/271(85.6%)	16/17(94.1%)	32/33(97%)	20/29(69%)	NS*†/0.020‡
SMA +	14/271(5.2%)	1/17(5.9%)	0	0	NS*†‡
Cirrhosis	147/277(53.1%)	10/17(58.8%)	19/33(57.6%)	21/31(67.7%)	NS*†‡

Data was shown as mean ± SD and/or median (range) and/or number/total (percent)

*AIH* Autoimmune hepatitis; *PBC* Primary biliary cholangitis; *CTD* Connective tissue disease; *TD* Thyroid dysfunction; *IgG/A/M* Immunoglobulin G/A/M; *WBC* White blood cell; *NE%* Neutrophil percentage; *MO%* Monocytes percentage; *Hb* Hemoglobin; *PLT* Platelet count; *MPV* Mean platelet volume; *Phos* Serum phosphorus; *CREA* Creatinine; *URCA* Uric acid; *GLU* Glucose; *ALT* Alanine aminotransferase; *AST* Aspartate aminotransferase; *TBIL* Total bilirubin; *DBIL* Direct bilirubin; *ALB* Albumin; *GLO* Globulin; *GGT* Gamma-glutamyl transferase; *ALP* Alkaline phosphatase; *TBA* Total bile acid; *TC* Total cholesterol; *TG* Triglycerides; *HDLC* High-density lipoprotein cholesterol; *LDLC* Low-density lipoprotein cholesterol; *PT* Prothrombin time; *APTT* Activated partial thromboplastin time; *INR* International normalized ratio; *TT* Thrombin time; *AFP* Alpha-1-fetoprotein; *ANA*+ Antinuclear antibody-positive; *SMA*+ Anti-smooth muscle antibody positive

NS, not significant (*P* > 0.05); Values in bold are significant (*P* < 0.05)

*P-value for AIH vs AIH-PBC

†*P*-value for AIH vs AIH-CTD

‡*P*-value for AIH vs AIH-TD

### Survival analysis of AIH complicated with immune diseases

**Fig. 1:**
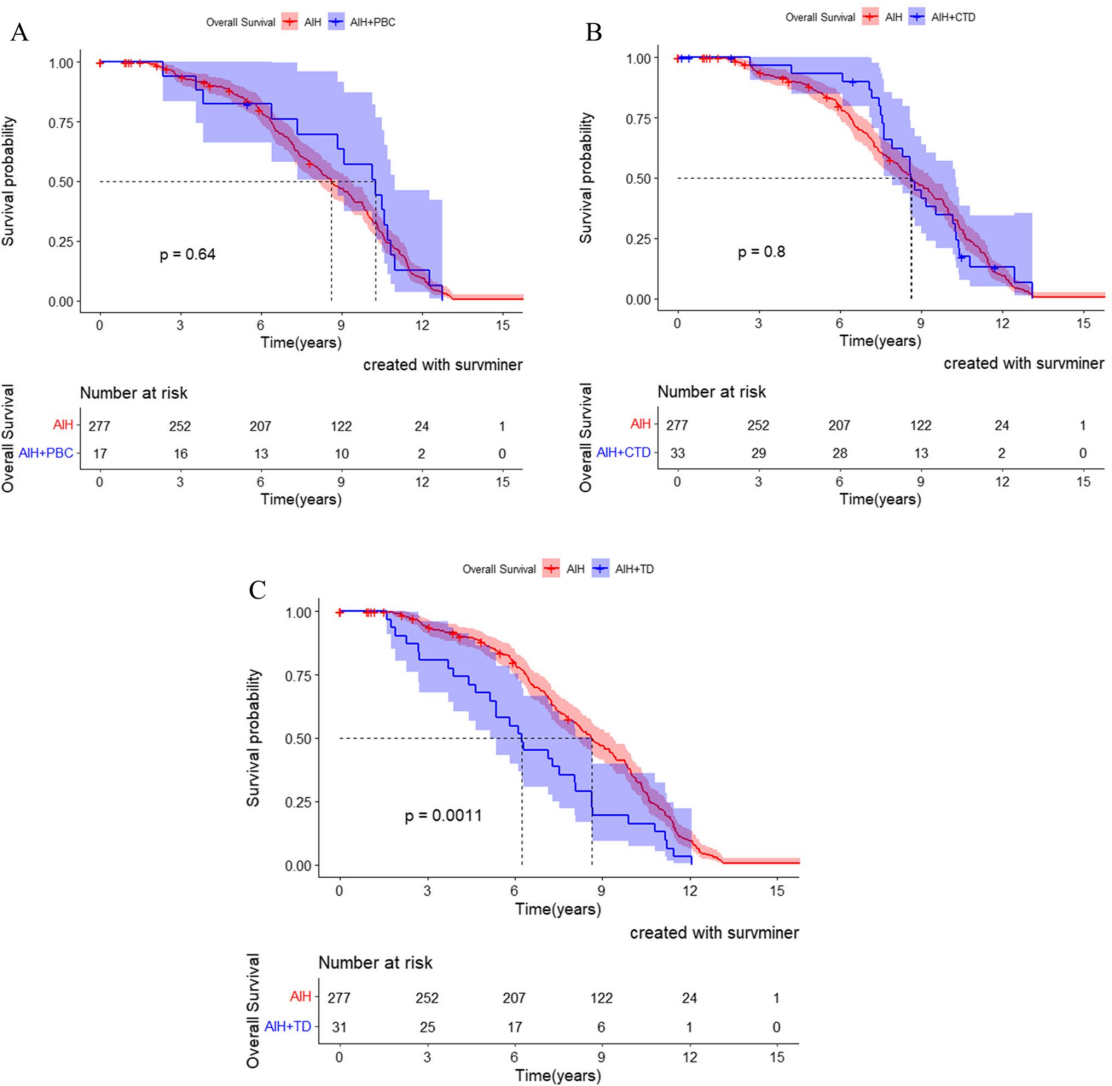
Survival curve of the patients with AIH, AIH-PBC, AIH-CTD and AIH-TD Abbreviations are same as in the text

As of December 31, 2021, the survival data of all patients were collected. In AIH group, 261 patients survived and 16 patients died from liver related diseases. The 3-year, 6-year and 9-year survival rates of AIH group were 90.9%, 74.7% and 44% respectively, and the median survival time was 8.4 years. In AIH-PBC group, 16 patients survived and 1 died. The 3-, 6- and 9-year survival rates were 94.1%, 76.5% and 58.8% respectively, and the median survival time was 10.2 years. In AIH-CTD group, 27 cases survived and 6 cases died. The 3-, 6- and 9-year survival rates were 87.9%, 84.8% and 39.4% respectively, and the median survival time was 8.4 years. In the AIH-TD group, 31 cases survived and none died. The 3-,6- and 9-year survival rates were 80.6%, 54.8% and 19.4% respectively, and the median survival time was 6.2 years. Therefore, Kaplan–Meier survival analysis showed that the overall survival time of AIH combined with TD was significantly shorter than that of AIH group (Fig. 1C, *P* = 0.0011), while the overall survival time of AIH-PBC or AIH-CTD was similar to that of AIH, with no statistical significance (Fig. 1A, *P* = 0.64 and Fig. 1B, *P* = 0.8, respectively).

### Univariate/multivariate cox regression analysis of prognostic factors of AIH patients

**Fig. 2:**
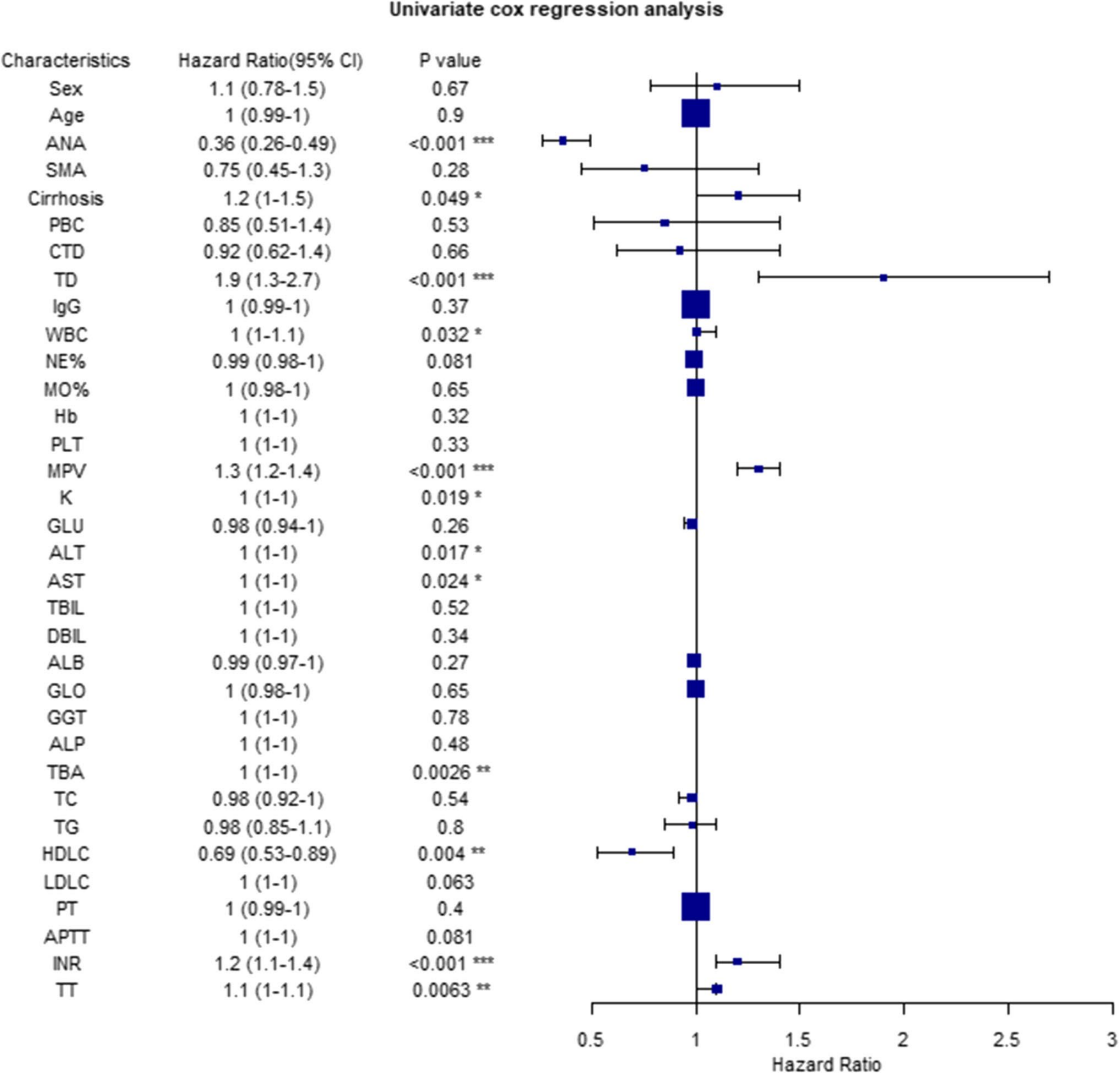
Univariate cox regression analysis of factors associated with reduced survival in AIH patients. Abbreviations are same as in the text. *P-value < 0.05; **P-value < 0.01; ***P-value < 0.001

Univariate analysis of prognostic factors of AIH patients by cox proportional risk model showed that ANA (*HR*: 0.36, 95% CI: 0.26–0.49, *P* < 0.001), liver cirrhosis (*HR*: 1.2, 95%CI: 1–1.5, *P* = 0.049), TD (*HR*: 1.9, 95%CI: 1.3–2.7, *P* < 0.001), WBC (*HR*: 1, 95%CI: 1–1.1, *P* = 0.032), MPV (*HR*: 1.3, 95%CI: 1.2–1.4, *P* < 0.001), serum K (*HR*: 1,95%) 95%CI: 1–1, *P* = 0.017), AST (*HR*: 1, 95%CI: 1–1, *P* = 0.024), TBA (*HR*: 1, 95%CI: 1–1, *P* = 0.0026), and HDL-C (*HR*: 0.69, 95%CI: 0.53–0.89, *P* = 0.004), INR (*HR*: 1.2, 95% CI: 1.1–1.4, *P* < 0.001) and TT (*HR*: 1.1, 95%CI: 1–1.1, *P* = 0.0063) were found to predict the survival time of AIH patients (Fig. 2).

**Fig. 3:**
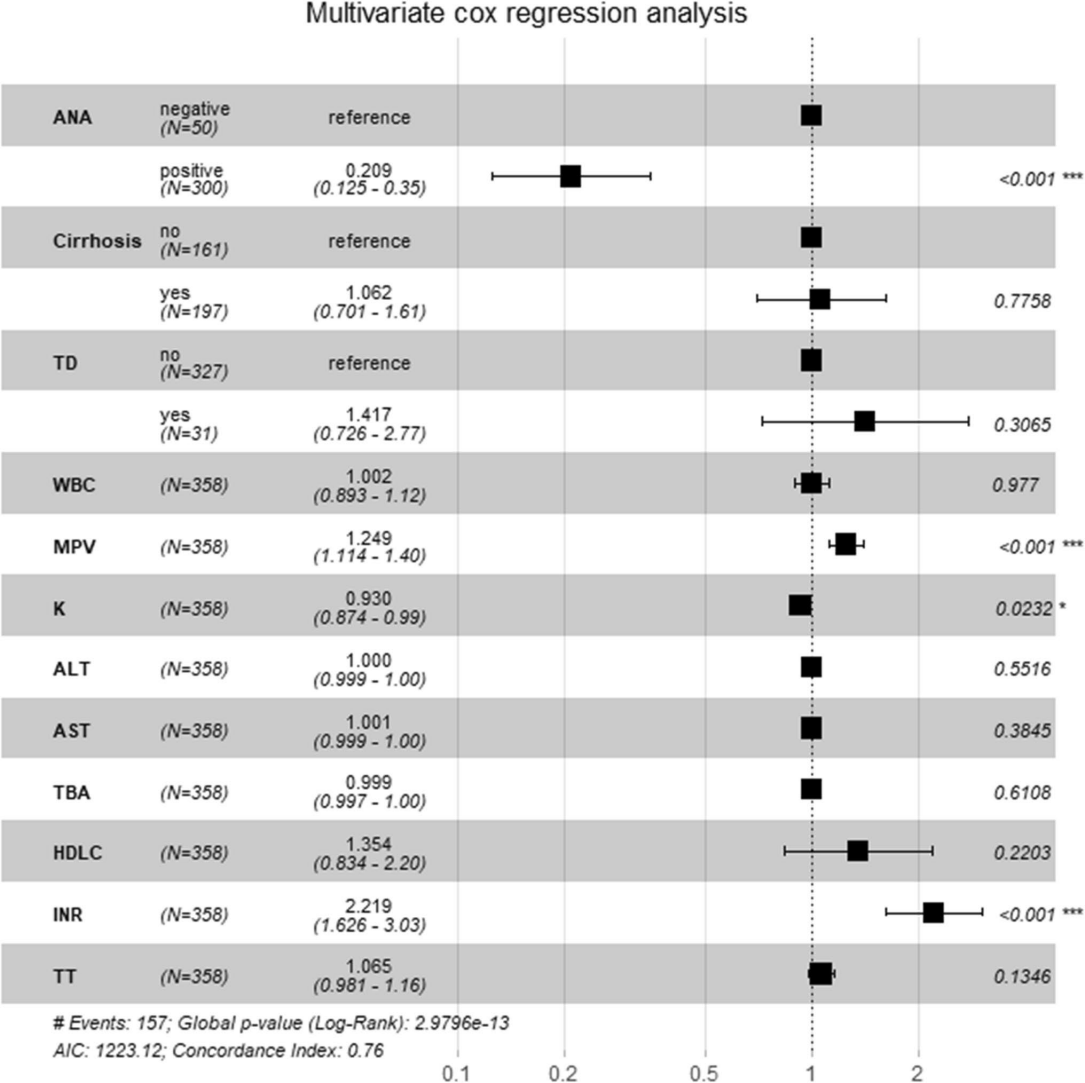
Multivariate cox regression analysis of factors associated with overall survival in AIH patients. Abbreviations are same as in the text. *P-value < 0.05; **P-value < 0.01; ***P-value < 0.001

Based on the result of univariate cox regression, multivariate cox regression analysis showed that ANA negative (*HR*: 0.21, 95%CI: 0.13–0.35, *P* < 0.001), MPV *(HR*: 1.25,95% CI: 1.11–1.4, *P* < 0.001), serum K (*HR*: 0.93, 95%CI: 0.87–0.99, *P* = 0.019) and INR (*HR*: 2.22, 95% CI: 1.63–3.03, *P* < 0.001) can be used as independent predictors of poor prognosis in AIH patients (Fig. 3).

### Kaplan–Meier survival curves to estimate the prognostic factors of AIH and comorbidity

**Fig. 4:**
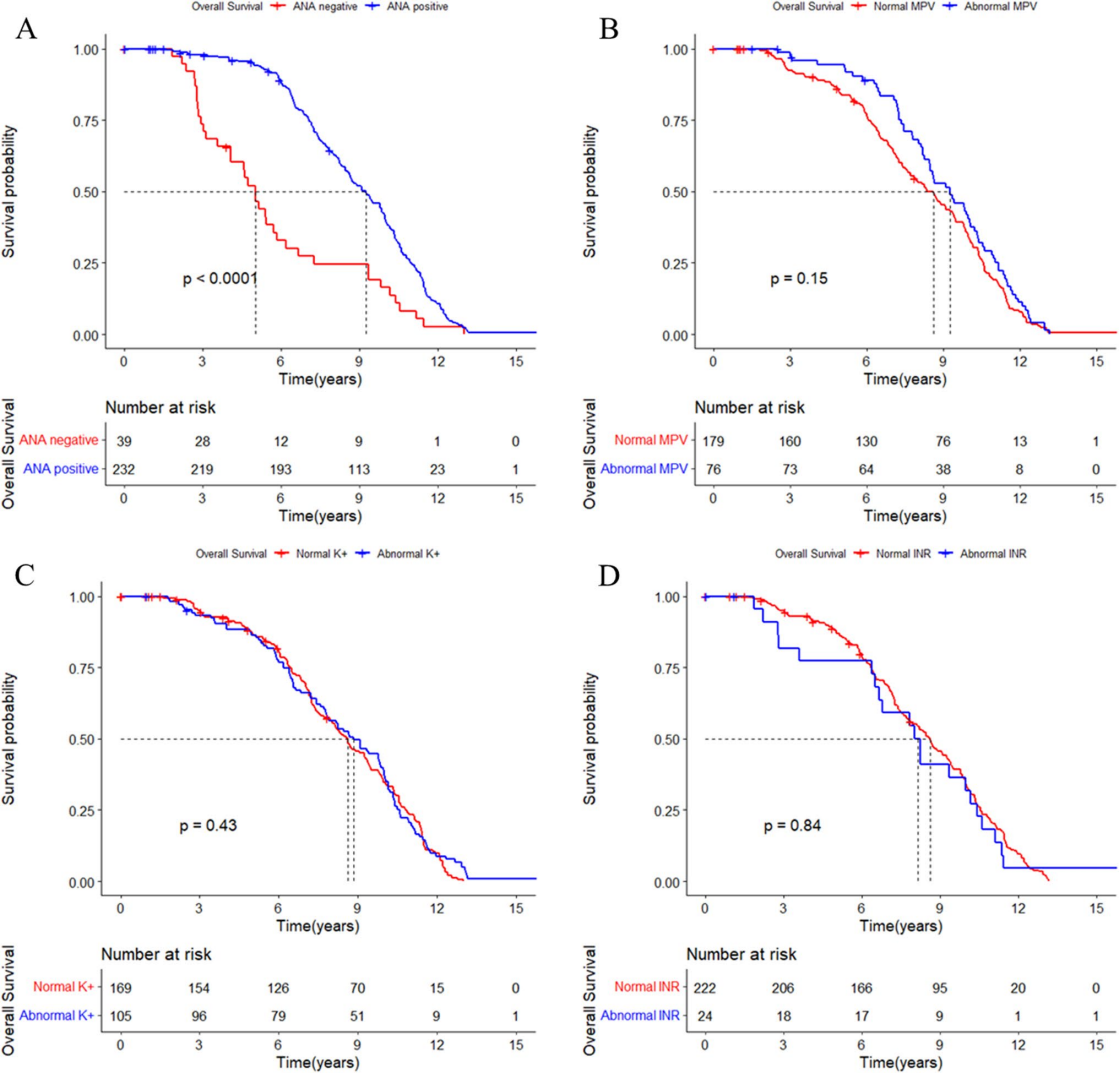
Kaplan–Meier analysis of factors associated with overall survival in AIH patients

**Fig. 5:**
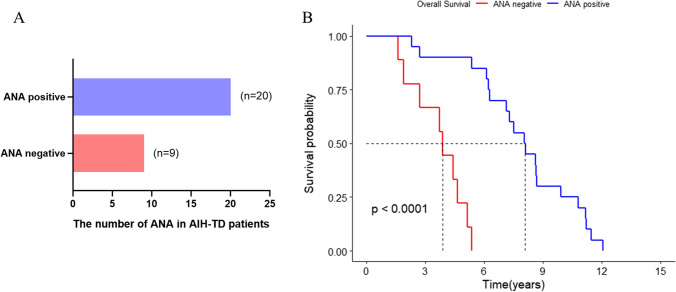
Kaplan–Meier analysis of ANA associated with overall survival in AIH-TD patients

As shown in Fig. 4A, it is worth mentioning that the survival time of ANA negative group was significantly shorter than the ANA positive group in AIH patients (*P* < 0.0001). There are no survival differences in groups sorted by MPV, serum K^+^ and INR (Fig. 4B–D). Considering the differences of ANA between AIH and AIH-TD patients, further Kaplan–Meier survival curves show that ANA negative can be used as an indicator to predict the poor prognosis of AIH and AIH-TD (Fig. 5).

## Discussion

This retrospective study specifically focuses on AIH complicated with immune diseases and compares incidence rate, clinical characteristics and prognosis. Three main findings arose from our study: (a) AIH is closely related to other immune diseases. (b) Patients with AIH-TD had significantly poor prognosis, and mean survival of patients in AIH-TD groups was found to be shorter than that of those with AIH. (c) It is worth noting that ANA negative can be used as independent indicators to predict the poor prognosis of AIH patients. To the best of our knowledge, this is a valuable study to provide basis for further mining the mechanism of AIH.

AIH is not only limited to intrahepatic immune injury, but also often associated with some extrahepatic immune diseases. Similar to the study of Italy and Germany, a study from the UK observed that 42% of AIH patients coexisted with extrahepatic immune diseases [13]. Some researchers presented that AIH often occurs in genetically susceptible individuals, causing intrahepatic or extrahepatic autoimmunity in individuals and families [23]. Another interesting finding was that 43% of AIH patients have extrahepatic immune diseases in their first-degree relatives, usually accompanied by thyroid diseases, diabetes and other disease types [24]. However, our data showed that only 26.5% of AIH patients were complicated with extrahepatic immune diseases, which was slightly lower than other studies.

Considering that immune diseases are related to low cognition in Asia, it further shows that immune diseases may be related to geographical location [25]. Interestingly, our research showed that in AIH complicated diseases, hypothyroidism (4.7%) is significantly higher than hyperthyroidism (0.8%). This is accordance with the previous research that AIH may affect thyroid function, and hypothyroidism is easier to be diagnosed than hyperthyroidism [26]. In view of few related studies on AIH complicated with immune diseases, it is necessary to carrying a large-scale cohort study. Considering that Beijing Ditan hospital is one of the largest clinical and research centers for liver diseases and infectious diseases in China, this cohort is highly representative. Therefore, the findings of this study were more closely resemble the real-world situation so as to further understand AIH complicated with intrahepatic/extrahepatic immune diseases.

Similar to the previous study, this study found that the weight of AIH patients is heavier than AIH-PBC group in this study, possibly due to malabsorption caused by the decrease of bile acid secretion in bile of PBC [27]. In addition, it was observed for the first time that ALT and AFP levels in AIH group were higher than AIH-PBC group, and IgM and ALP levels were significantly lower in this study (Table 2). It can be inferred from two assumptions. Firstly, AIH is insidious, and about 7% of patients have progressed to cirrhosis at the time of diagnosis [28]. Considering that AIH and PBC had different injury position, AIH is mainly localized in liver parenchyma injury, while bile duct injury caused by immune disorder in PBC, which bring the diversity of liver function. Secondly, the immune response of AIH-PBC patients is stronger, resulting in higher IgM than AIH patients, which was consistent with Ma's research [29]. In addition, we also found that AIH-CTD patients showed a lower level of serum K and MPV (Table 2), which may be due to the involvement of kidney and hematopoietic function in CTD patients, resulting in renal tubular acidosis [30]. In all, the clinical features of AIH complicated with different immune diseases are various.

As AIH may occur on the rise, it is clear that the prognosis of AIH is noteworthy. The other interesting finding in our study is that ANA, MPV, serum K^+^ and INR can be used as independent prognostic factors of AIH (Figs. 2 and 3), which is consistent with Yamamoto's research conclusion that INR is related to the occurrence of death outcomes in AIH patients [31]. A study from Japan showed that age and stage IV of liver fibrosis are independent risk factors affecting the overall survival of AIH [32]. Another national study from Denmark found that male and cirrhosis were unfavorable factors for the prognosis of AIH [33]. In contrast, Joshita's research showed that ANA has no significant difference in clinical manifestation, histopathology or disease outcome of AIH, but ALP ≥ 500 or GGT ≥ 200 may be an independent risk factor for AIH recurrence [34]. Therefore, it is indispensable for AIH patients to evaluate prognosis by paying attention to clinical indicators.

Moreover, in this study, the proportion of ANA-positive in AIH-TD patients was significantly less than that of AIH patients (69% vs. 85.6%, *P* = 0.02). Interestingly, the survival time of AIH-TD patients was significantly shorter than that of AIH patients (Fig. 1, *P* = 0.001), while the overall survival of AIH-PBC and AIH-CTD patients was not significantly different from AIH patients. Another interesting finding was the survival time of ANA negative group was significantly shorter than that of the ANA positive group in AIH and AIH-TD (Figs. 4, 5), which is rarely reported. Furthermore, some studies also reported that ANA was related to the prognosis of immune diseases [35, 36], such as systemic lupus erythematosus, dermatomyositis. However, whether or not ANA could predict the prognosis outcome of AIH remains to be further studied.

There are some limitations in this study: Firstly, this study only included AIH patients who were hospitalized in a tertiary hospital specializing in the treatment of liver diseases, which may lead to bias in the inclusion of patients. Secondly, because this study is a single center and the sample size is relatively small, there is no statistical significance in some clinical features. Finally, because this study is a retrospective design, it is inevitable that some indicators cannot be included in the data collection, and there are some uncontrolled biases. Therefore, further multicenter studies are needed, which need a large enough sample size and a long follow-up period to detect the significant correlation between the results.

## Conclusions

Our data showed that AIH was closely related to other immune diseases and about 26.5% of AIH patients had at least one immune disease in a cohort from China. It was also found that TD coexisted with AIH could impaired patients’ survival significantly. Moreover, we found that ANA negative can be used as an independent indicator to predict the poor prognosis of AIH and comorbidity patients. Therefore, identifying unique clinical features and biochemical manifestations to locate specific involved organs may be helpful for early diagnosis, treatment and better prognosis of AIH.

## Data Availability

Data available on request from the authors.

## Abbreviations

AIH: Autoimmune hepatitis
PBC: Primary biliary cholangitis
CTD: Connective tissue disease
TD: Thyroid dysfunction
IgG/A/M: Immunoglobulin G/A/M
WBC: White blood cell
NE%: Neutrophil percentage
MO%: Monocytes percentage
Hb: Hemoglobin
PLT: Platelet count
MPV: Mean platelet volume
Phos: Serum phosphorus
CREA: Creatinine
URCA: Uric acid
GLU: Glucose
ALT: Alanine aminotransferase
AST: Aspartate aminotransferase
TBIL: Total bilirubin
DBIL: Direct bilirubin
ALB: Albumin
GLO: Globulin
GGT: Gamma-glutamyl transferase
ALP: Alkaline phosphatase
TBA: Total bile acid
TC: Total cholesterol
TG: Triglycerides
HDLC: High-density lipoprotein cholesterol
LDLC: Low-density lipoprotein cholesterol
PT: Prothrombin time
APTT: Activated partial thromboplastin time
INR: International normalized ratio
TT: Thrombin time
AFP: Alpha-1-fetoprotein
ANA+: Antinuclear antibody-positive
SMA+: Anti-smooth muscle antibody positive
HR: Hazard ratio
SD: Standard deviation
CI: Confidence interval
